# Diabetic cardiomyopathy: effects of fenofibrate and metformin in an experimental model – the Zucker diabetic rat

**DOI:** 10.1186/1475-2840-8-16

**Published:** 2009-03-24

**Authors:** Fabien Forcheron, Alexandra Basset, Pauline Abdallah, Peggy Del Carmine, Nicolas Gadot, Michel Beylot

**Affiliations:** 1 EA4173-ERI22 Agressions vasculaires et réponses tissulaires Faculté Rockefeller, UCBLyon1 and INSERM, Lyon, France; 2 ANIPATH Faculté RTH Laennec, UCBL1, Lyon, France; 3 ANIPHY Faculté Rockefeller, UCBLyon1, Lyon, France; 4 EA4173-ERI 22, Faculté RTH Laënnec, Rue G Paradin, 69008, Lyon, France

## Abstract

**Background:**

Diabetic cardiomyopathy (DCM) contributes to cardiac failure in diabetic patients. It is characterized by excessive lipids accumulation, with increased triacylglycerol (TAG) stores, and fibrosis in left ventricle (LV). The mechanisms responsible are incompletely known and no specific treatment is presently defined. We evaluated the possible usefulness of two molecules promoting lipid oxidation, fenofibrate and metformin, in an experimental model of DCM, the Zucker diabetic rat (ZDF).

**Methods:**

ZDF and controls (C) rats were studied at 7, 14 and 21 weeks. After an initial study at 7 weeks, ZDF rats received no treatment, metformin or fenofibrate until final studies (at 14 or 21 weeks). C rats received no treatment. Each study comprised measurements of metabolic parameters (plasma glucose, TAG, insulin levels) and sampling of heart for histology and measurements of TAG content and relevant mRNA concentration.

**Results:**

ZDF rats were insulin-resistant at 7 weeks, type 2 diabetic at 14 weeks and diabetic with insulin deficiency at 21 weeks. Their plasma TAG levels were increased. ZDF rats had at 7 weeks an increased LV TAG content with some fibrosis. LV TAG content increased in untreated ZDF rats at 14 and 21 weeks and was always higher than in C. Fibrosis increased also moderately in untreated ZDF rats. Metformin and fenofibrate decreased plasma TAG concentrations. LV TAG content was decreased by metformin (14 and 21 weeks) and by fenofibrate (14 weeks). Fibrosis was reduced by fenofibrate only and was increased by metformin. Among the mRNA measured, fenofibrate increased Acyl-CoA Oxidase mRNA level, metformin decreased Acyl-CoA Synthase and increased AdipoR1 and pro-inflammatory mRNA levels.

**Conclusion:**

Fenofibrate had favourable actions on DCM. Metformin had beneficial effect on TAG content but not on fibrosis. PPARα agonists could be useful for the prevention and treatment of DCM.

## Background

Diabetes mellitus increases the risk of cardiovascular diseases and the incidence of heart failure [1,2]. This heart failure may result from hypertension and/or from accelerated development of coronary atherosclerosis [3]. Diabetic patients can also develop a specific cardiomyopathy called diabetic cardiomyopathy (DCM) [4,5]. Mechanisms responsible for DCM are still poorly understood but abnormalities in lipid metabolism with increased accumulation in left ventricle (LV) of intra-cellular lipids, demonstrated by the increase in triglycerides (TAG) content [6,7], play an important role [3,8-13]. These abnormalities in lipid metabolism could contribute in particular to the apoptosis of cardiomyocytes and the development of fibrosis [3,8,10]. This role of excess lipid stores (lipotoxicity [14]) is supported by experimental models. Accumulation of lipids in cardiomyocytes, through overexpression of long-chain acyl-CoA synthase (ACS) or lipoprotein-lipase (LPL) [12,15] or inhibition of fatty acid oxidation [16], induces cardiomyopathy. On the contrary, augmented efflux of lipids from heart reduces its TAG content and the development of cardiomyopathy [17,18]. The mechanisms leading to increased heart lipids accumulation and TAG stores during diabetes are still debated. Fatty acid oxidation is increased in diabetic heart [10,19] but the rise in fatty acid uptake could be still more important resulting in accumulation of lipids [20]. The rise in uptake could result from increased availability of plasma substrates (TAG and non esterified fatty acids (NEFA)) during diabetes, increased expression of molecules involved in fatty acid uptake [10,21] or an association of both.

No treatment of DCM has been defined besides treatment of diabetes. PPARγ agonists reversed in Zucker diabetic rats (ZDF) heart lipid accumulation and development of DCM [8]. However thiazolidinediones expand body fluids volume [22], may have adverse effects on cardiac function and one should be cautious in their utilization [23]. PPARα agonists could be useful: they lower plasma TAG concentrations [24] and reduce TAG content in skeletal muscle [25] and heart [26]. In addition PPARα agonists can prevent LV diastolic dysfunction in OLETF rats [27]. However cardiac-restricted overexpression of PPARα in mice induces cardiac lipid accumulation and cardiomyopathy [9,28]. Although there may be differences between the effects of overexpression of a transcription factor in a given tissue and the effects of whole body activation of this transcription factor expressed at physiological level, these observations [9,28] raises serious concern on the use of PPARα agonists in the presence of DCM. Clarifying this point is all the more important that PPARα agonists are frequently used in the treatment of hyperlipidemia in diabetic subjects. Therefore, we investigated in an experimental model of DCM, the ZDF rat [8,29] the effects of fenofibrate, a PPARα agonist used in the treatment of hypertriglyceridemia [24], on two main manifestations of DCM, TAG accumulation and fibrosis in LV. Metformin is widely used in the treatment of type 2 diabetes. It stimulates fatty acid oxidation through the AMP activated kinase (AMPK) [30] and can reduce lipids accumulation in the skeletal muscles of ZDF rats [31]. Therefore metformin could also lower intra-cellular lipids content in LV and opposes the development of DCM. We also investigated its effects on the development of DCM in ZDF rats.

## Methods

### Protocols

Male ZDF rats (fa/fa) (n = 35) and their control littermates (controls C, +/+) (n = 15) (Charles River, L'Arbresle, France) arrived at the age of six weeks and were housed in an animal facility with controlled temperature (22 ± 1°C) and a 12 h light/dark cycle (light on at 7:00 AM). Throughout the study they had free access to water and food. All rats received the diet (Purina 5008, IPS, London, UK) (protein 26.8%, carbohydrate 56.4% (91% starch, 9% simples carbohydrates), fat 16.7% of caloric value) recommended for the development of diabetes in male ZDF rats. Water and food intake, body weight were recorded five time/week. A first metabolic investigation was performed in all rats after one week of acclimation (7 weeks old). Thereafter, five rats of the control and ZDF groups were sacrificed for blood and tissue sampling. The remaining control rats were divided in two groups (five rats each); one was sacrificed at the age of 14 weeks after a second metabolic investigation, the other had metabolic investigations at 14 and 21 weeks before sacrifice at 21 weeks. ZDF rats were divided in three groups of 10 rats. One group received only the Purina 5008 diet (ZDF group); the other groups received also fenofibrate (100 mg/kg/day, mixed with diet, ZDF+F group) or metformin (300 mg/kg/day, mixed with diet, ZDF+M group), doses comparable to those used in previous studies in rats [32]. Fenofibrate or metformin administration started only once the first metabolic investigation was completed and was continued until the final sacrifice. Five rats of each group were sacrificed at 14 weeks after a second metabolic investigation; the remaining five rats were investigated at 14 and 21 weeks before sacrifice at 21 weeks. Experiments were conducted according to the French laws and regulation for experiments in animals.

### Metabolic investigations

Each investigation comprised blood sampling (tail vein) in the fed state for measurement of plasma insulin, NEFA and TAG concentrations and of blood glucose level (One Touch Ultra, Life Technology, Issy-Les-Moulineaux, France), In addition, an insulin tolerance test (ITT) was performed in control and ZDF rats at 7 weeks, during the first metabolic investigation. Food was removed at 07:00 AM. Insulin (1 unit/kg) was injected intra-peritoneally at 01:00 PM. Blood glucose was measured before and 15, 30, 45, 60, 90 and 120 min after insulin injection.

Five rats of each group were sacrificed at 7, 14 and 21 weeks. Food was removed the morning at 08:00 AM. Rats were anesthetized at 02:00 PM (pentobarbital IP 60 mg/kg). Blood (inferior vena cava) was collected, centrifuged and plasma stored at -20°C until analysis. Heart was removed, washed with cold isotonic saline and weighed. LV was quickly collected and weighted. One part was flash frozen in liquid nitrogen and stored at -80°C until analysis and one part fixed (4% paraformaldehyde) for histological analysis.

### Analytical procedures

Plasma NEFA and TAG were measured by enzymatic methods [24] and insulin by ELISA (Cristal Chem, Downers Grove, USA). For measurements of LV TAG concentrations, 100 mg of tissue were homogenized in chloroform/methanol (1:2, v:v). The chloroform phase was collected, washed with water and dried under nitrogen. Extracted lipids were dissolved in propanol for enzymatic determination of TAG concentration.

LV RNAs were purified (TRIZOL, Invitrogen, Cergy-Pontoise, France) and treated with DNase. Total RNA was reverse transcripted (Superscript II (Invitrogen) and random hexamers). Real time PCR was performed in a MyIQ thermal cycler (Biorad, Marnes La Coquette, France) using iQSYBR green Supermix. Samples were run in duplicate along with dilutions of known amounts of target sequence for quantification of initial cDNA copies. Results are expressed as the target over 18S RNA concentration ratio. Primer sequences are given in Additional file 1. Cross sections of the LV fixed with formaldehyde and embedded with paraffin were stained with Sirius Red. For each LV sample, collagen density was evaluated in two non-consecutive sections in the epicardial, middle and endocardial parts of the ventricle as the Sirius Red positive area over total area ratios Images were acquired with a Coolscope microscope (Nikon, Tokyo; Japan) (magnification × 20) and analyzed blinded with respect to the appartenance to the different groups.

### Statistics

Results are shown as mean ± sem. Intra-groups comparisons of values obtained at 7, 14 and 21 weeks for the various groups of rats (i.e. control, ZDF, ZDF+M and ZDF+F groups) were performed by one way ANOVA followed by the Newman-Keuls procedure to locate the differences. Between groups comparisons of the values obtained for each metabolic investigation (at 7, 14 or 21 weeks) were performed by one way ANOVA followed by the Newman-Keuls test. P < 0.05 was considered as significant. We used GraphPad Prism 4.0 software (GraphPad, San Diego, CA, USA).

## Results

### Food intake and body weight (Table 1)

**Table 1:** Evolution of food intake and of body weight in control rats and in ZDF rats receiving or not fenofibrate (F) or metformin (M).

Food intake (g/day)	Control	ZDF	ZDF + F	ZDF + M
7 weeks	18.5 ± 0.4	27.5 ± 0.7 ***	26.7 ± 0.7***	26.3 ± 0.6***

14 weeks	26.5 ± 0.9	46.9 ± 2.4***	43.5 ± 2.4***	47.1 ± 2.1***

21 weeks	26.6 ± 1.0	46.5 ± 3.0 ***	46.9 ± 3.7***	43.8 ± 2.2***

Body weight (g)				

7 weeks	188 ± 4	231 ± 5***	253 ± 11***	228 ± 8**

14 weeks	325 ± 14	345 ± 5	314 ± 5$	360 ± 13

21 weeks	409 ± 9	361 ± 6**	326 ± 8***$	398 ± 25

Results are shown are mean and sem. * p < 0.05, ** p < 0.01, *** p < 0.001 vs the corresponding control; $ p < 0.05 vs the corresponding ZDF group.

ZDF rats ate more than control rats throughout the study (p < 0.001). Fenofibrate and metformin did not modify food ingestion. At 7 weeks ZDF rats had a higher body weight than control rats (p < 0.01). There was no difference in body weight at 7 weeks between ZDF rats who received fenofibrate or metformin and those who received no treatment. Control rats gained weight between 7 and 14 weeks (p < 0.01) and between 14 and 21 weeks (p < 0.01). ZDF rats gained weight between 7 and 14 weeks (p < 0.01), but less than control rats and there was no further gain at 21 weeks. Their body weight was comparable to the one of control rats at 14 weeks and lower at 21 weeks (p < 0.01). Rats receiving fenofibrate gained less weight than the ZDF group and at 21 weeks their weight was less than the one of the control and ZDF groups (p < 0.05). Metformin had no significant effect on body weight of ZDF rats.

### Insulin and metabolites concentrations (Table 2, Table 3 and Table 4)

**Table 2:** Evolution of plasma glucose and insulin (fed state) in control rats and in ZDF rats receiving or not fenofibrate (F) or metformin (M).

Glucose mM	Control	ZDF	ZDF + F	ZDF + M
7 weeks	7.4 ± 0.3	7.3 ± 0.3	7.6 ± 0.2	7.4 ± 0.3

14 weeks	7.0 ± 0.4	30.1 ± 0.9***	31.5 ± 0.5***	31.6 ± 0.6***

21 weeks	7.6 ± 0.3	30.2 ± 0.2 ***	33.2 ± 0.2***	34.2 ± 0.4***

Insulin ng/l				

7 weeks	4.7 ± 0.7	16.0 ± 3.0**	12.7 ± 0.8**	12.4 ± 1.1**

14 weeks	6.8 ± 1.1	4.3 ± 1.0	5.1 ± 0.7	6.3 ± 0.7

21 weeks	8.5 ± 0.5	<0.5 **	<0.5 **	<0.5 **

Results are shown are mean and sem. ** p < 0.01, *** p < 0.001 vs the corresponding control

**Table 3:** Evolution of plasma TAG and NEFA (measured in the post-absorptive state) in control rats and in ZDF rats receiving or not fenofibrate (F) or metformin (M).

TAG mM	Control	ZDF	ZDF + F	ZDF + M
7 weeks	0.47 ± 0.04	4.12 ± 0.48 ***		

14 weeks	0.50 ± 0.07	3.79 ± 0.40 ***	2.97 ± 0.77 **$	1.48 ± 0.42 *$$

21 weeks	0.54 ± 0.04	5.83 ± 1.01 ***	3.26 ± 0.40 ***$	2.40 ± 0.50 **$

NEFA μM				

7 weeks	595 ± 71	491 ± 28		

14 weeks	512 ± 59	1150 ± 95 **	985 ± 68 **	1052 ± 59 **

21 weeks	485 ± 63	1495 ± 152 ***	1310 ± 152 ***	1158 ± 125 ***

Results are shown are mean and sem. ** p < 0.01, *** p < 0.001 vs the corresponding control; $ p < 0.05, $$ p < 001 vs the corresponding ZDF group. These parameters were measured only in rats sacrificed for tissue sampling (n = 5 for each group), therefore they were measured at 7 weeks only in a group of 5 control and ZDF rats.

**Table 4:** Evolution of plasma TAG and NEFA (measured in the fed state) in control rats and in ZDF rats receiving or not fenofibrate (F) or metformin (M).

TAG mM	Control	ZDF	ZDF + F	ZDF + M
7 weeks	0.89 ± 0.06	2.85 ± 0.39 ***	4.09 ± 0.44 ***	3.32 ± 0.29 ***

14 weeks	1.34 ± 0.08	10.40 ± 1.10 ***	6.50 ± 1.19 ***$	8.60 ± 1.07 ***

21 weeks	1.50 ± 0.12	9.86 ± 0.63 ***	12.10 ± 1.42 ***	13.20 ± 0.57 ***

NEFA μM				

7 weeks	550 ± 62	435 ± 43	541 ± 64	523 ± 89

14 weeks	541 ± 64	1062 ± 103 ***	869 ± 107 ***	1098 ± 123 **

21 weeks	416 ± 27	824 ± 115 **	1121 ± 145 ***	1211 ± 104 ***

Results are shown are mean and sem** p < 0.01, *** p < 0.001 vs the corresponding control group; $ p < 0.05 vs the corresponding ZDF group.

#### - Glucose and insulin levels (table 2)

ZDF rats had at 7 weeks comparable blood glucose concentrations but higher plasma insulin (p < 0.01) than control rats, indicating the presence of insulin-resistance. This resistance to insulin was confirmed by the ITT (see additional file 2). Diabetes developed in all ZDF rats around the tenth week as shown by the increase in water consumption and urinary output. In all ZDF rats at 14 and 21 weeks, glucose was above 25 mM. Plasma insulin decreased to values comparable to those of control rats at 14 weeks and below the detection level at 21 weeks. Neither fenofibrate nor metformin modified these evolutions.

#### - Plasma lipid concentrations (Table 3 and Table 4)

At 7 weeks control and ZDF rats had comparable NEFA concentrations whereas TAG were higher in ZDF rats (fed and post-absorptive states). There were no differences in these parameters (fed state) between ZDF rats receiving thereafter fenofibrate, metformin or no treatment. Plasma lipid concentrations were unchanged at 14 and 21 weeks in control rats. NEFA concentrations were much higher in diabetic ZDF rats, both at 14 or 21 weeks and in the fed and post-absorptive state, than both in corresponding control rats of 14 or 21 weeks and in ZDF rats before the appearance of diabetes. Neither fenofibrate nor metformin modified plasma NEFA levels in ZDF rats. Plasma TAG were always higher in ZDF than in control rats at 14 and 21 weeks; Compared to those of ZDF rats at 7 weeks, these values were higher in the fed state (p < 0.01) but not in the post-absorptive one. Fenofibrate and metformin decreased plasma TAG levels in the post-absorptive state, when most TAG originate from liver TAG synthesis and secretion, with a more marked effect of metformin particularly at 14 weeks. On the contrary, in the fed state, when most TAG originate from the absorption of ingested lipids, metformin had no effect and fenofibrate had only a moderate TAG lowering effect at 14 weeks. These differences between effects in the fed and post-absorptive states suggest that both metformin and fenofibrate acted on plasma TAG levels more by lowering liver TAG secretion than by increasing plasma TAG clearance.

### Heart and LV weight and TAG content (figure 1)

**Figure 1:**
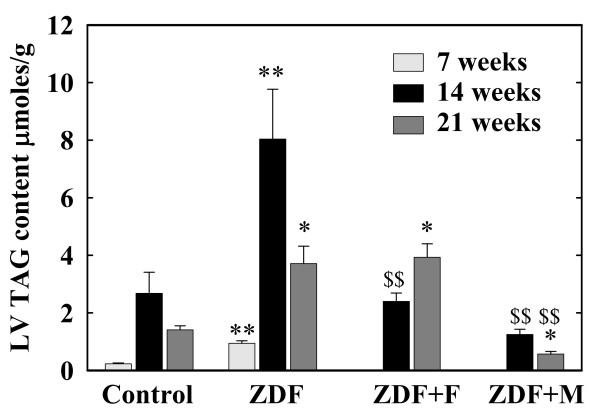
**Concentration of triacylglycerols (TAG) in the left ventricle (LV) of Control and ZDF rats**. ZDF rats were untreated (ZDF) or received fenofibrate (ZDF+F) or metformin (ZDF+M) after the first metabolic investigation at the age of 7 weeks. These parameters were measured only in rats sacrificed for tissue sampling (n = 5 for each group), therefore they were measured at 7 weeks only in a group of control and ZDF rats.* p < 0.05, ** p < 0.01 vs the corresponding control group; $$ p < 0.01 versus the corresponding untreated ZDF groups. For the sake of clarity, differences within the control group and the ZDF group between values at 7, 14 and 21 weeks are not indicated; these differences are indicated in the results section.

At 7 weeks ZDF rats had moderate and parallel increases (p < 0.05 vs controls) in heart (880 ± 22 vs 806 ± 19 mg) and LV weights (604 ± 7 vs 566 ± 7 mg). These weights were slightly decreased in ZDF rats at 14 and 21 weeks. The LV over heart weight ratios were unchanged except for a moderate increase in ZDF rats at 14 weeks (0.712 ± 0.018 vs 0.629 ± 0.024 in control rats p < 0.05). Neither fenofibrate not metformin modified these parameters in ZDF rats.

7-week ZDF rats had already higher LV TAG content than control rats (p < 0.01). In both groups this content increased at 14 and 21 weeks (p < 0.01) with values always higher (p < 0.05 or 0.01) in ZDF rats. Fenofibrate normalized this TAG content at 14 weeks (p < 0.01 vs untreated ZDF) but had no effect at 21 weeks. Metformin effect was more marked with significant decreases (p < 0.01) at 14 and 21 weeks. At 21 weeks, TAG content of LV was even lower in ZDF rats receiving metformin than in control rats (p < 0.05).

### Fibrosis

**Figure 2:**
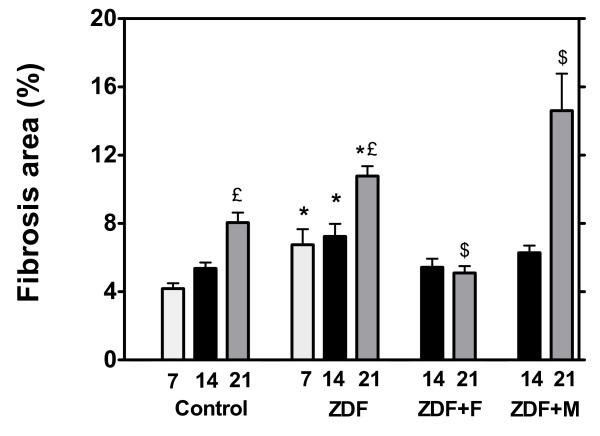
**Histological quantification of fibrosis (expressed as per cent of total areas) in control and ZDF rats**. ZDF rats were untreated (ZDF) or received fenofibrate (ZDF+F) or metformin (ZDF+M) after the first metabolic investigation at the age of 7 weeks. These parameters were measured only in rats sacrificed for tissue sampling (n = 5 for each group), therefore they were measured at 7 weeks only in a group of control and ZDF rats. * p < 0.05 vs the corresponding control group; € p < 0.05 vs the 7 week value of the group; $ p < 0.05 vs the corresponding untreated ZDF group.

Histological studies showed that collagen deposition (both interstitial and perivascular) was increased (p < 0.05) in 7-week ZDF rats compared to 7-week control rats (figure 2, see additional file 3). Collagen deposition increased moderately with age in control rats (p < 0.05 at 21 weeks). It increased more in untreated ZDF rats with values always higher than in controls (p < 0.05). This fibrosis was reduced in the ZDF group receiving fenofibrate with a significant decrease at 21 weeks (p < 0.05). On the contrary, despite the marked decrease in TAG accumulation, fibrosis was not reduced by metformin and was even increased at 21 weeks (p < 0.05).

### mRNA levels in left ventricle (figures 3 and 4)

**Figure 3:**
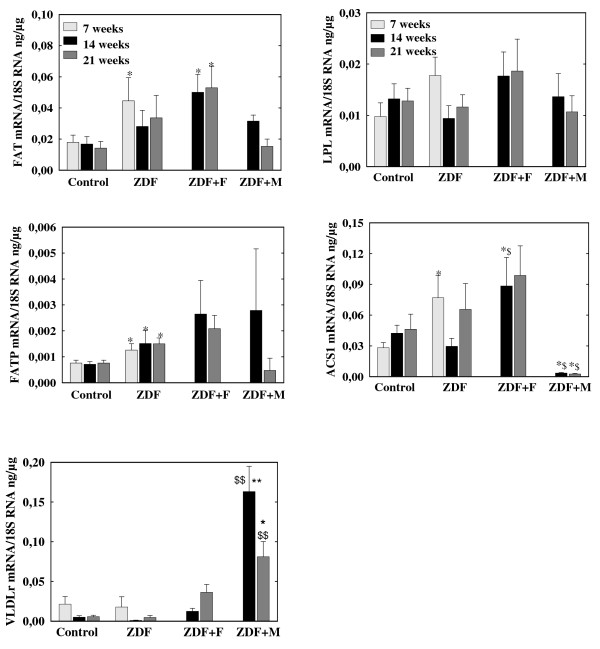
**mRNA concentrations of of genes controlling fatty acids uptake (FAT, FATP, VLDLr, LPL) and activation (ACS1) in left ventricles of control and ZDF rats**. ZDF rats were untreated (ZDF) or received fenofibrate (ZDF+F) or metformin (ZDF+M).* p < 0.05, ** p < 0.01 vs the corresponding control group, $ p < 0.05, $$ p < 0.01 vs the corresponding untreated group. For the sake of clarity, differences within the control group and the ZDF group between values at 7, 14 and 21 weeks are not indicated

**Figure 4:**
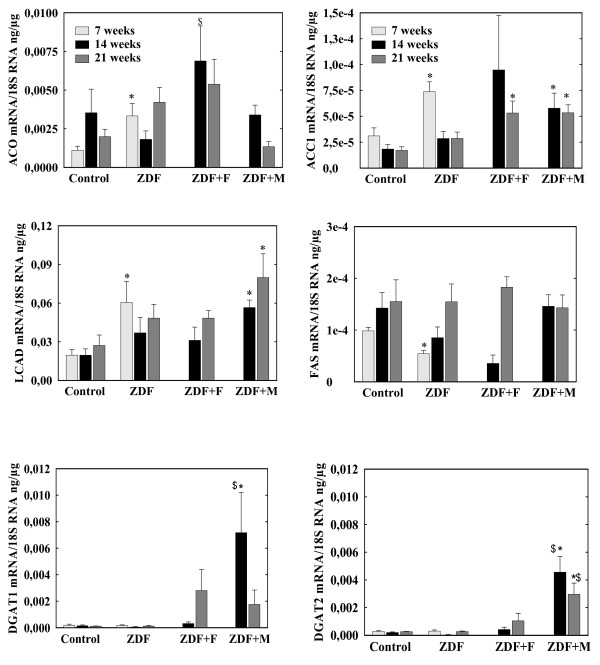
**mRNA of genes controlling fatty acids synthesis (ACC1, FAS), esterification into TAG (DGAT1 and 2) and oxidation (ACO, LCAD)**. ZDF rats were untreated (ZDF) or received fenofibrate (ZDF+F) or metformin (ZDF+M).* p < 0.05 vs the corresponding control group, $ p < 0.05 vs the corresponding untreated group. For the sake of clarity, differences within the control group and the ZDF group between values at 7, 14 and 21 weeks are not indicated

#### a) Fatty acids metabolism (figure 3 and 4)

7-week ZDF rats had moderate increases (p < 0.05) in mRNA concentrations of Fatty Acid Translocase (FAT), Fatty Acid Transport Protein (FATP) and Acyl-CoA synthase1 (ACS1), all molecules involved in fatty acids uptake or activation. Acetyl-CoA carboxylase2 (ACC2) mRNA levels were comparable in ZDF and control rats (data not shown). ACC1 mRNA levels were increased in ZFD rats (p < 0.01) but mRNA level of Fatty Acid Synthase (FAS), the other enzyme controlling *de novo *lipogenesis was decreased (p < 0.01). Expressions in ZDF rats of diacylglycerol-acyl transferase 1 and 2 (DGAT1 and DGAT2), involved in TAG synthesis, was unchanged. Long chain-CoA Acyl dehydrogenase (LCAD) and Acyl-CoA Oxidase (ACO), two enzymes of fatty acid oxidation were increased (p < 0.05).

mRNA levels of control rats at 14 and 21 weeks were comparable to values at 7 weeks despite a trend for lower VLDLr mRNA. In ZDF rats there was a decrease in ACC1 mRNA concentrations at 14 and 21 weeks (p < 0.01 vs 7 weeks) and a trend for lower values in FAT, VLDLr, ACS1 and LCAD mRNA. There was always a trend for higher FAT and LCAD mRNA in ZDF than in control rats at 14 and 21 weeks but the only significant difference was the persistence of increased FATP mRNA (p < 0.05).

**Figure 5:**
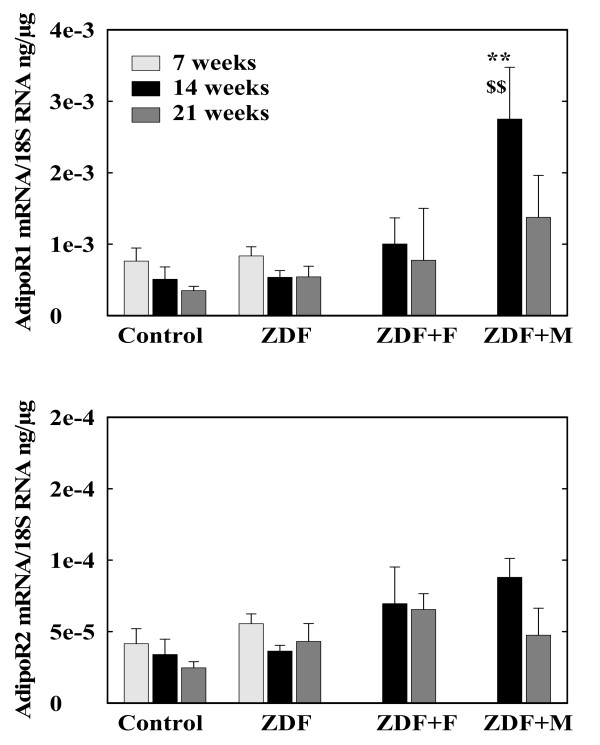
**mRNA concentrations of adiponectin receptors (AdipoR1 and AdipoR2) in left ventricles of control and ZDF rats**. ZDF rats were untreated (ZDF) or received fenofibrate (ZDF+F) or metformin (ZDF+M). ** p < 0.01 vs the corresponding control group, $$ p < 0.01 vs the corresponding untreated group.

Fenofibrate induced a trend for an increase in FAT (whose mRNA levels were higher than in control rats p < 0.05), FATP, LPL, VLDLr and DGAT1 mRNAs both at 14 and 21 weeks but the only significant effects were increases in ACS1 and ACO (p < 0.05 vs untreated ZDF) at 14 weeks. Metformin increased VLDLr (p < 0.01 at 14 and 21 weeks), DGAT1 (p < 0.05 at 14 weeks) and DGAT2 (p < 0.05) expressions but induced a trend for lower FAT and FATP mRNAs at 21 weeks and it clearly decreased ACS1 mRNA at 14 and 21 weeks (p < 0.01). It induced also a trend for increased LCAD mRNA, that was higher than in control rats at 14 and 21 weeks (p < 0.05);

Since adiponectin stimulates lipid utilization [33] including in cardiomyocytes [34] and there are data suggesting that PPARα can modify the expression of its receptors, AdipoR1 and R2, [35] we measured also the expression of these receptors in LV (figure 5). AdipoR1 and R2 mRNA levels were comparable in control and untreated ZDF rats and were not modified by fenofibrate. However, metformin increased the expression of AdipoR1 in ZDF at 14 weeks (p < 0.01 vs untreated ZDF and vs Control) and induced a trend for higher values at 21 weeks.

#### b) Fibrosis and inflammation (figures 6)

**Figure 6:**
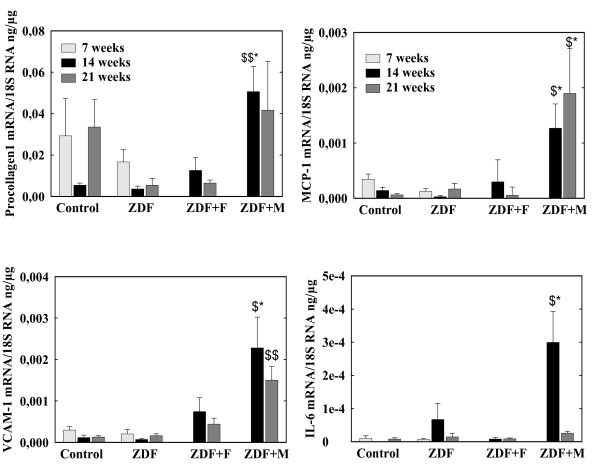
**mRNA concentrations of procollagen-1, MCP-1, VCAM-1 and IL-6 in left ventricles of control and ZDF rats**. ZDF rats were untreated (ZDF) or received fenofibrate (ZDF+F) or metformin (ZDF+M).* p < 0.01 vs the corresponding control group, $ p < 0.05, $$ p < 0.01 vs the corresponding untreated group.

We observed no difference in procollagen3 mRNA between ZDF and control rats and no modifications during the administration of fenofibrate or metformin (data not shown). Procollagen1 mRNA level was not higher in ZDF rats than in control rats and was unchanged in the fenofibrate treated group but was increased by metformin administration (p < 0.01 at 14 weeks). The expressions of VCAM-1, Il-6 and MCP-1 were comparable in control and untreated ZDF rats and unchanged by fenofibrate but all increased during the administration of metformin. Endothelin-1 expression was comparable en control and ZDF rats and unchanged by fenofibrate or metformin administration (data not shown).

## Discussion

ZDF rats had at 7 weeks of age, in agreement with previous studies [8], heart abnormalities with increased heart and LV weights, increased TAG content and already presence of some fibrosis. TAG content and fibrosis increased further in more aged rats (14 and 21 weeks old). TAG stores increased also in control rats and some fibrosis was also present in 14 and 21 weeks old control rats, but to a less extent than in ZDF rats. Our results confirm that heart abnormalities appear early in this model of DCM; they are present at the initial stage of insulin-resistance (7 weeks old rats) and are aggravated when diabetes is present (14 and 21 weeks).

TAG accumulation can result from a shift in the fate of fatty-acyl-CoA toward esterification with a corresponding decrease in oxidative rate, or from an imbalance between cellular fatty acid uptake and oxidation, with a more important increase in uptake than in oxidation, in the absence of any absolute decrease in lipid oxidation. Zhou et al [8] suggested that fatty acid oxidation was decreased in the heart of ZDF rats. However, this is difficult to reconcile with the augmented fatty acid oxidation classically reported in heart during insulin-resistance and diabetes [10,19,36]. Our finding of increased ACO and LCAD mRNA levels in 7 weeks old ZDF rats supports indeed an enhanced lipid oxidation rate. The increased mRNA concentrations for FAT, FATP and ACS1 in 7 weeks old ZDF rats also support the idea that, in addition to an increased availability of circulating lipid substrates (at least TAG), the ability of heart to take up and activate fatty acids is increased. In addition, fatty acid transporters are relocated from an intra-cellular pool to the plasma membrane in heart of Zucker rats and this contributes to an enhanced fatty acid uptake [21]. Therefore, there is evidence that in 7-weeks ZDF rats, in a situation of insulin-resistance, LV TAG accumulation is induced by an inappropriate increase in fatty acid uptake resulting itself from increased substrate availability and enhanced capacity for uptake. An additional potential source of fatty acids for TAG synthesis is *in situ de novo *lipogenesis. Both FAS and ACC1 mRNA were present in heart and ACC1 mRNA concentrations were increased in 7 weeks old ZDF rats. However, FAS mRNA was decreased in these rats. FAS activity is low in heart [37] and cardiac ACC1 activity is decreased in insulin resistant and diabetic heart [36]. Therefore, *de novo *lipogenesis is a quantitatively minor pathway in heart and is not enhanced by insulin-resistance and diabetes. The respective roles of increased circulating lipid substrates availability and of heart ability to take up fatty acid could be different in 14 and 21 weeks old ZDF rats, when overt diabetes is present, from their role in 7-weeks ZDF rats. Only FATP mRNA remained increased in ZDF rats once diabetes was present. Therefore, although it remains possible that the preferential relocation of fatty acids transporters to plasma membrane described in Zucker rats [21] was always present, our results suggest that in 14 and 21 weeks old ZDF rats the increased uptake and accumulation of lipids by heart result mainly of the large increases in plasma NEFA and TAG concentrations.

We investigated the potential usefulness for the prevention or treatment of DCM of fenofibrate and metformin. Fenofibrate had a mild beneficial action since it reduced fibrosis and, in 14 weeks old rats only, LV TAG content. These results are consistent with previous reports showing that PPARα activation decreased skeletal muscle TAG content [25] and also heart TAG content of db/db mice [26]. They are also consistent also with studies showing an anti-fibrotic effect of fenofibrate in several models of hypertensive cardiomyopathy [38-40]. They do not support the concerns for the use of fibrates in humans raised by the adverse effects in mice of heart-specific overexpression of PPARα [9,28,41]. Fenofibrate increased at 14 weeks the expression of ACO but also of ACS1 and induced a trend for higher expression of FAT, FATP and LPL suggesting a parallel stimulation of pathways for fatty acids uptake, activation and oxidation. These results on gene expression agree with those obtained in cultured cardiomyocytes [42,43]. Aasum et al found no effect of *in vivo *administration of another PPARα activator on the expression of target genes of PPARα in heart of db/db mice [44]; however they measured mRNA levels only after *ex vivo *experiments with isolated working heart. In our experiments, it is difficult to determine whether the balance between uptake and activation on one hand and oxidation on the other was modified. It is probable that a main effect of fenofibrate was to reduce plasma TAG concentration and thus an important source of fatty acids uptake by heart. This would agree with the fact that the plasma TAG lowering effect of fenofibrate was more marked at 14 weeks, when LV TAG content was reduced, than at 21 weeks, when it did not reduce LV TAG stores. Whatever the exact mechanism, our data show no deleterious effects but rather beneficial actions of a PPARα agonist on heart TAG accumulation in this model of DCM. Fibrosis was also reduced by fenofibrate. This anti-fibrotic effect of PPARα activators was also observed in hypertensive cardiomyopathy and was ascribed in these models to an anti-inflammatory action [38-40]. We found in the present model of DCM no increased expression of pro-inflammatory molecules genes, or any reduction of these expressions by fenofibrate. Fibrosis in DCM is considered to result from the apoptosis of cardiomyocytes induced by the accumulation of cytotoxic lipid molecules [8]. Therefore, the present anti-fibrotic action of fenofibrate in the present study is probably related to the reduction in lipid accumulation.

Metformin was more effective than fenofibrate in reducing LV TAG content. This could result also from the decrease in plasma TAG levels, at least in the post-absorptive state. However, an unexpected finding is the clear decrease in ACS1 mRNA observed with metformin. ACS1 is the main ACS expressed in heart. ACS2 is expressed at much lower level and ACS3, 4 and 5 are expressed at negligible levels [45-47]. ACS1 expression is regulated in liver, particularly by nutritional factors [47]. In rodents heart, ACS1 expression is increased par PPARα agonists [48], in agreement with the present results, but we know no previous study showing modifications of ACS expression by metformin or other activators of AMPk, in heart or in other tissues. Inhibition of ACS1 expression by metformin is difficult to reconcile with the idea that metformin, through AMPk activation [30], stimulates fatty acids oxidation, since decreased fatty acids activation would reduce the availability of substrates for lipid oxidation. Surprisingly, metformin did not reduce fibrosis despite the decrease in LV TAG content. Moreover the expressions of procollagen 1 and of pro-inflammatory and adhesion molecules were increased. Thus, there is in this situation a discrepancy between lipids accumulation on one hand, fibrosis and inflammation on the other. This is surprising since, although metformin did not decrease circulating levels of C reactive protein and cell adhesion molecules in subjects with impaired glucose tolerance [49], it reduced the TNFα-induced activation of NFκB and secretion of VCAM-1 and MCP-1 in vascular endothelial cells [50]. The mechanisms behind this persistent fibrosis and increased inflammation in heart during metformin treatment remain unclear. They could be related to another unexpected finding, the stimulation by metformin of heart AdipoR1 expression. This increase in adiponectin receptor expression could result in enhanced actions of adiponectin on heart. It could contribute to the decrease in TAG content since adiponectin stimulates lipid oxidation [33]. With respect to fibrosis and inflammation, correlative studies in non-alcoholic steatohepatitis suggest that adiponectin has anti-fibrotic effects [51] and adiponectin is considered to have rather an anti-inflammatory actions. However, several studies suggest that adiponectin, at least its globular form, activates NFκB and the expression of proinflammatory and adhesion molecules genes in macrophages, endothelial cells and cardiac fibroblasts [52-54]. In addition, it stimulates the proliferation of cardiac fibroblasts and collagen synthesis [52]. These actions seem mediated mainly through AdipoR1, the receptor with the highest affinity for globular adiponectin [55]. Thus metformin-induced increase in AdipoR1 expression could have contributed to fibrosis and to the increase of pro-inflammatory and adhesion molecules genes expression.

## Conclusion

we found, in the model used, that fenofibrate had no adverse but rather favourable actions on DCM with mild reduction in TAG accumulation and fibrosis. These results strongly suggest that PPARα activators should not have deleterious effects on myocardium in subjects with DCM and could even be useful in such patients. Metformin had a clear lowering effect on heart TAG content but did not reduce fibrosis and stimulated the expression of proinflammatory and adhesion molecule genes. Further studies are needed to clarify these effects of metformin, the mechanisms behind them and to determine whether they are present in humans or not.

## Competing interests

The authors declare that they have no competing interests.

## Authors' contributions

FF realized most of the mRNA measurements, participated in the experimental part of the study, the statistical analysis and drafting of the manuscript. AB, PA and PC realized most of the experimental part of the study. PA was also involved in the measurement of various plasma and left ventricle metabolites concentrations and in mRNA measurements. NG realized histological studies. MB was responsible for the conception, general design and realization of the study, participated in its realization and drafted the manuscript.

## Supplementary Material

Additional file 1 **Table.** Primers used for qPCR determination of mRNA concentrations. Click here for file

Additional file 2 **Figure S1.** Insulin tolerance test in 7-week old controls (open circles, n = 15) and ZDF (closed circles, n = 35) rats. The results obtained in the different groups of ZDF rats (receiving thereafter no treatment, receiving thereafter metformin or fenofibrate) were comparable. Click here for file

Additional file 3 **Figure S2.** Histological study of left ventricles od control and DZF rats. Representative samples of left ventricles of 7-week old control (A) and ZDF rats (B), of 14-week and 21-week old Control (C and G respectively) and ZDF rats untreated (D and H), or treated by fenofibrate (E and I) or metformin (F and J). Staining is with red Sirius. Fibrosis appears in red. Click here for file
