# Supplementary material for: Diabetic cardiomyopathy: effects of fenofibrate and metformin in an experimental model – the Zucker diabetic rat
Source: Cardiovasc Diabetol. 2009 Mar 24;8:16. doi: 10.1186/1475-2840-8-16 (PMC2664796; doi:10.1186/1475-2840-8-16)
Supplement: Additional file 1 — Table. Primers used for qPCR determination of mRNA concentrations. [file 1475-2840-8-16-S1.doc]

Table 1. Primers used for real-time PCR determination of mRNA levels:

| Name | Forward primer | Reverse primer | Size of fragment |
| --- | --- | --- | --- |
| AdipoR1 | ctggactattcagggattg | acacagacgatggagaggta | 115 |
| AdipoR2 | atgtttgccacccctcagta | agcctatctgccctatggt | 139 |
| ACO | atggctacgggttacatgcc | ttgtccatcttcaggtagcc | 142 |
| ACS1 | tcagactccgctccatc | gcccatccaggtcattc | 115 |
| ACC1 | caacgcaggcatcagaa | caagtattccacagtccc | 138 |
| ACC2 | tcctgcccactttcttc | cggtgctgtaggctgtt | 102 |
| DGAT1 | aagtatggcatcctggtgga | caggcgcttctcaatctgaa | 138 |
| DGAT2 | gtgggtcctatccttcc | acgcccaagaaaggt | 134 |
| FAS | ggtgctacccattcgtg | ggatgtatcattcttggactt | 115 |
| FAT | aggaagtggcaaagaat | tgaaggctcaaagatgg | 155 |
| IL-6 | ccttcttgggactgatg | ctggtctgttgtgggtg | 96 |
| FATP | cctgcggcttcaaca | tcagtggctccatcgt | 84 |
| LCAD | cccgatgttctcattctg | ccattcttcgtggtagg | 164 |
| LPL | cctgaagacacagctgagga | cacccaactctcatacattc | 141 |
| MCP-1 | aatgggtccagaagtac | tcagatttatgggtcaa | 130 |
| Procollagen1 | accagcctcgtccacag | cgggcagggttctttcta | 124 |
| VCAM-1 | gtttgcctcgctaagtt | gatggtgggttctttcg | 105 |
| VLDLr | tctggagttcctagctcat | ccagtgaatttattggcacc | 108 |
| 18S | tgaggccatgattaagaggg | agtcggcatcgtttatggtc | 190 |
