# Supplementary material for: Diabetic cardiomyopathy: effects of fenofibrate and metformin in an experimental model – the Zucker diabetic rat
Source: Cardiovasc Diabetol. 2009 Mar 24;8:16. doi: 10.1186/1475-2840-8-16 (PMC2664796; doi:10.1186/1475-2840-8-16)
Supplement: Additional file 2 — Figure S1. Insulin tolerance test in 7-week old controls (open circles, n = 15) and ZDF (closed circles, n = 35) rats. The results obtained in the different groups of ZDF rats (receiving thereafter no treatment, receiving thereafter metformin or fenofibrate) were comparable. [file 1475-2840-8-16-S2.doc]

Supplemental figure 1: Insulin tolerance test in 7-week old controls (open circles, n=15) and ZDF (closed circles, n=35) rats. The results obtained in the different groups of ZDF rats (receiving thereafter no treatment, receiving thereafter metformin or fenofibrate) were comparable.
