# Supplementary material for: Diabetic cardiomyopathy: effects of fenofibrate and metformin in an experimental model – the Zucker diabetic rat
Source: Cardiovasc Diabetol. 2009 Mar 24;8:16. doi: 10.1186/1475-2840-8-16 (PMC2664796; doi:10.1186/1475-2840-8-16)
Supplement: Additional file 3 — Figure S2. Histological study of left ventricles od control and DZF rats. Representative samples of left ventricles of 7-week old control (A) and ZDF rats (B), of 14-week and 21-week old Control (C and G respectively) and ZDF rats untreated (D and H), or treated by fenofibrate (E and I) or metformin (F and J). Staining is with red Sirius. Fibrosis appears in red. [file 1475-2840-8-16-S3.doc]

Supplemental figure 2: Representative samples of left ventricles of 7-week old control (A) and ZDF rats (B), of 14-week and 21-week old Control (C and G respectively) and ZDF rats untreated (D and H), or treated by fenofibrate (E and I) or metformin (F and J). Staining is with red Sirius. Fibrosis appears in red.

**A
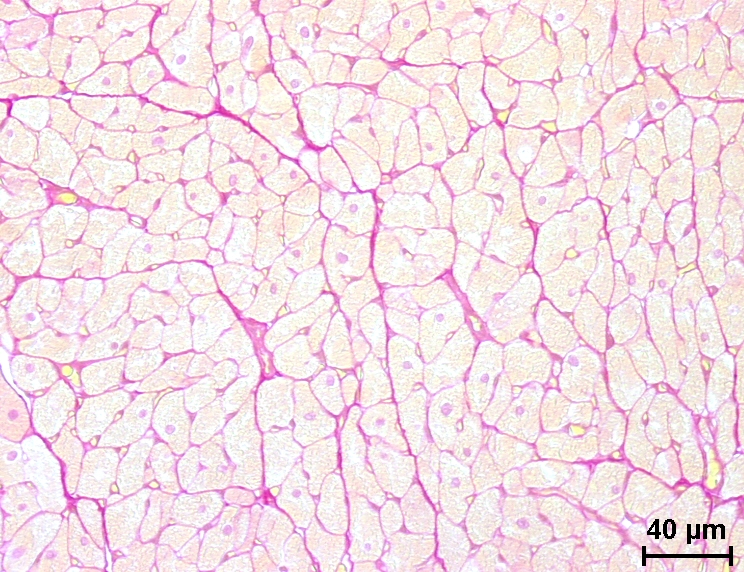
 B
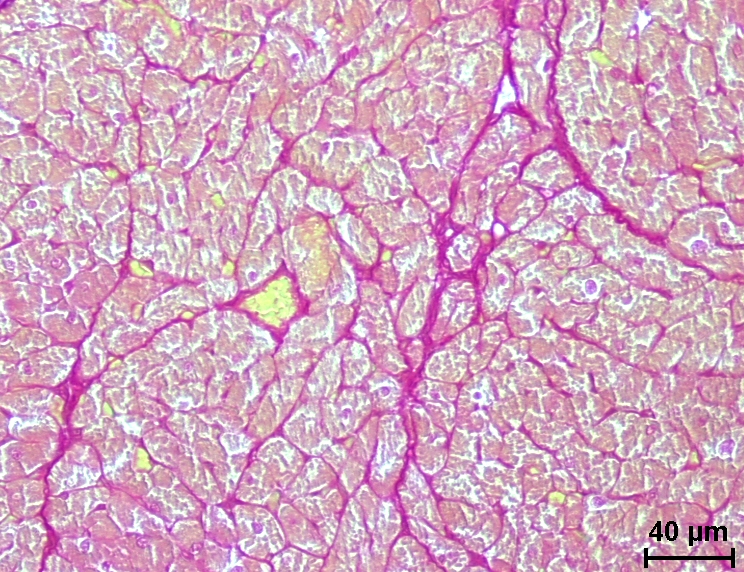
**

**C
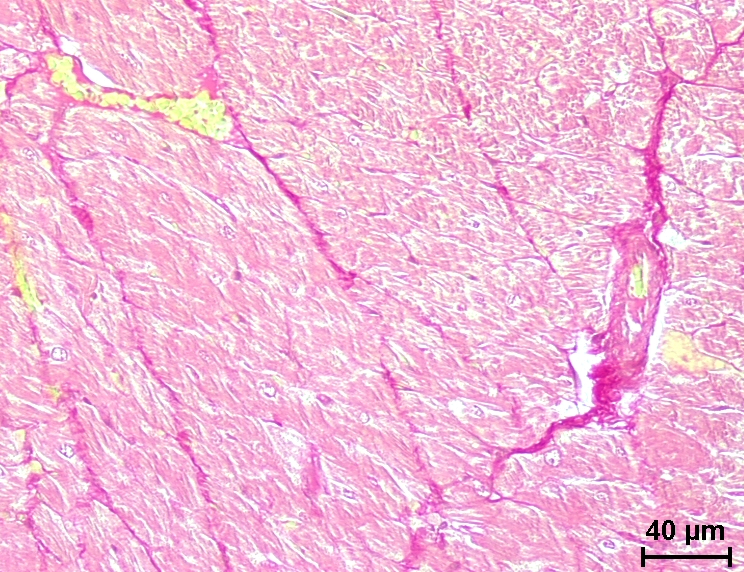
D
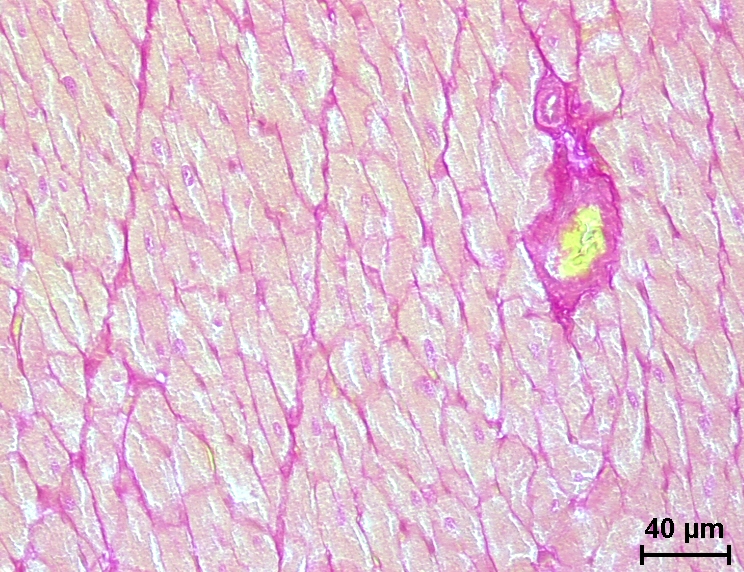
**

**E
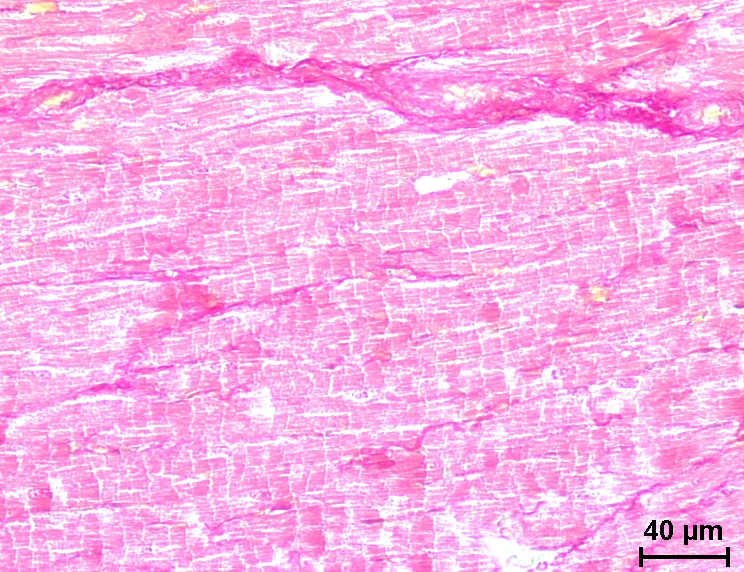
F
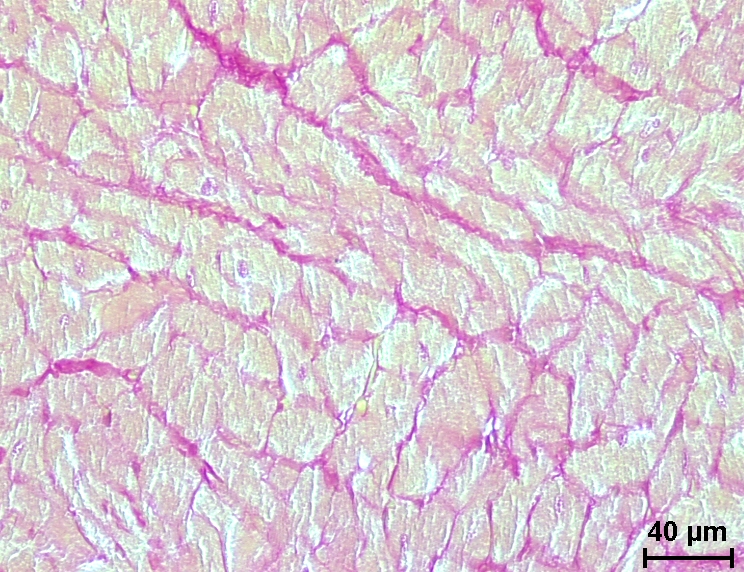
**

**G
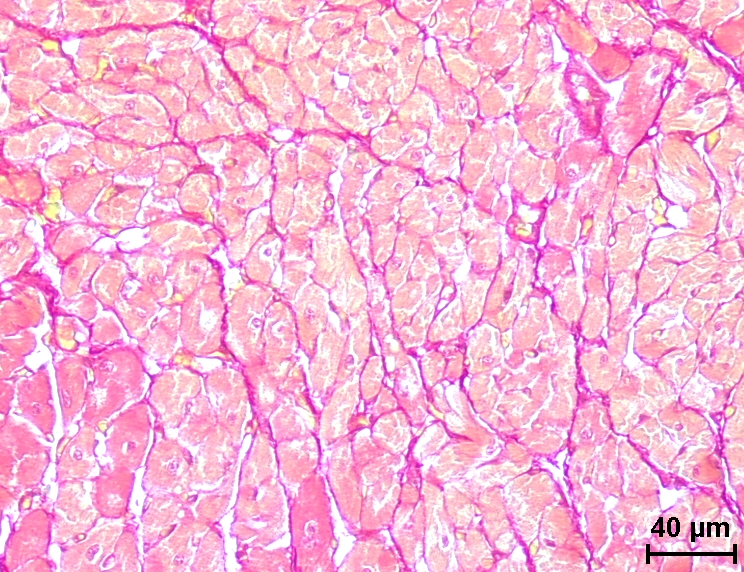
H
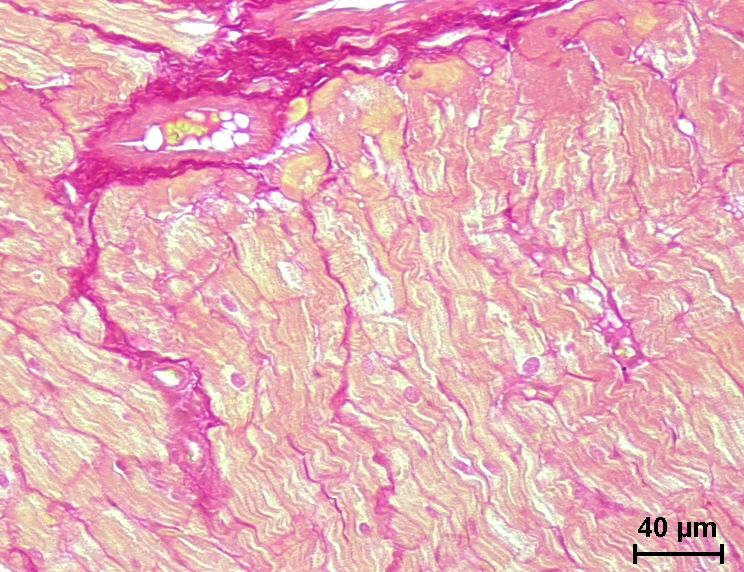
**

**I
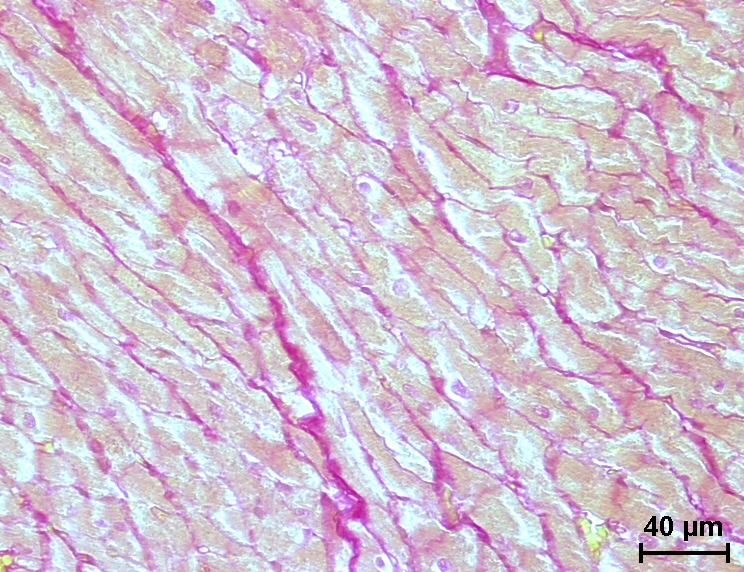
J
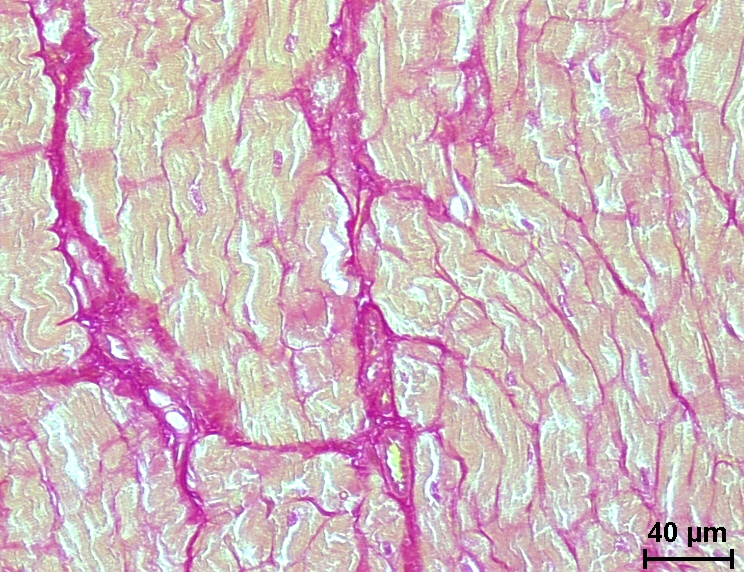
**
